# Reciprocating trust in Registered Reports

**DOI:** 10.1038/s44271-024-00101-9

**Published:** 2024-05-24

## Abstract

*Communications Psychology* celebrates the publication of the journal’s first two Stage-2 Registered Reports. We encourage researchers regardless of career stage to consider the format and highlight some considerations for PhD students and early career researchers.

**Figure:**
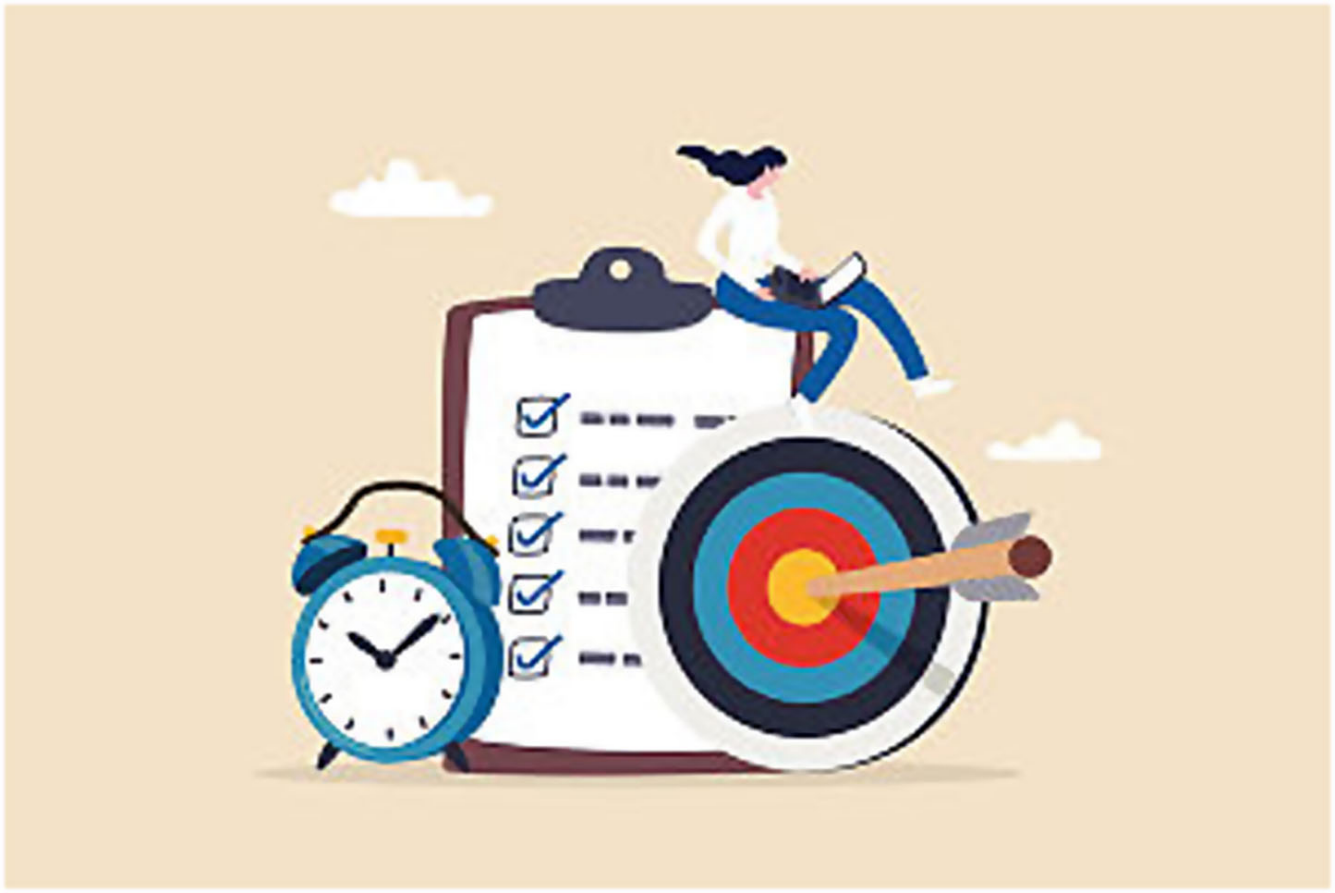
http://picscout.springernature.com/fotoweb/archives/5000-All-Files/2024/04/22/AdobeStock_578977889.ai.info#c=%2Ffotoweb%2Farchives%2F5000-All-Files%2F%3Fq%3Dplanning. © Nuthawut / Stock.adobe.com

Registered Reports are an article format where a detailed research plan is peer-reviewed and accepted in principle for publication before collection of primary data begins. The format was introduced in Cortex in 2013^1^ and rapidly gained in popularity.

*Communications Psychology* encourages Registered Report submissions for all types of confirmatory research, not only for replication studies^2^. So far, about one in 15 research submissions that the journal has received have been Registered Reports. Indeed, the first paper to ever receive acceptance-in-principle at *Communications Psychology* was a Registered Report^3^.

We are now proud to join the ranks of journals who not only offer Registered Reports but have published completed works.

The first of these two publications^4^, consists of a combination of a replication of previous work and an extension of the research question in a modified paradigm. The work by Engeler et al. replicates the finding that when humans refrain from checking the personal cost of helping others, it signals trustworthiness. The authors then tested whether this finding would generalize to the context of punishment. The data here are more complex; there is no statistically significant evidence that deliberation over the personal costs of punishment affects trustworthiness.

The second Registered Report, authored by Offer et al.^5^, examines whether participants seek or avoid information on whether they are receiving fair or unfair offers, and if knowing about the (un)fairness of an offer then affects acceptance rates. More than half of participants purposefully ignored whether they had received an unfair offer; these deliberately ignorant participants then also tended to accept the offers at a much higher rate.

These two studies are exemplary for the type of confirmatory research that lends itself to Registered Reports. Though it may be reassuring to note that Registered Reports work for a variety of confirmatory research proposals^6^, the question remains: do Registered Reports also work for researchers at various career stages?

“I would definitely recommend PhD students take this approach, although of course it is a lot easier with the support of their supervisor – mine was very much in favour of it.”

We asked the first authors of the Registered Reports^4,5^, both graduate students working towards their PhDs, what they thought. “I would definitely recommend PhD students take this approach,” confirmed Engeler, “although of course it is a lot easier with the support of their supervisor – mine was very much in favour of it.”

One of the most common hesitations that PhD students express is that the delay between submitting the proposal and obtaining the data will harm their graduation prospects. Indeed, the timespan from submission to acceptance is longer than for standard Research Articles, because the time period that is spent collecting and analyzing the data, as well as the time that it takes to write up the Results and Discussion count into this period. And comparing the number of rounds of peer-review for Research Articles and Registered Reports, the latter likely require one additional round (two before data is collected, one afterwards).

However, this does not necessarily mean that the time from conceptualization of a research project to its final publication is longer for Registered Reports. For example, peer-review of standard Research Articles frequently involves requests for further data collection when authors judge their work to be already complete. This is not the case for Registered Reports. Moreover, because standard Research Articles are much more frequently rejected by journals after data collection, authors regularly face delays in the form of multiple rounds of review at different journals. In comparison, the time span from the completion of data analysis to publication for Registered Reports is fast, involves a single journal, and typically the same referees.

The authors of both Registered Reports agreed that the delay created by peer review prior to data collection pays off in other ways. As Offer explains, “we could incorporate [peer reviewers’] feedback effectively at the time when it was most helpful”. Engeler highlighted that she could work on other projects while she was waiting during the peer-review process and that she might recommend the format especially for PhD students in the beginning or middle stages of their programme, rather than towards the end.

“We could incorporate feedback effectively at the time when it was most helpful.”

A related consideration for researchers facing the job market is that Registered Report protocols are deposited in a repository when the journal issues acceptance-in-principle, i.e., just before data collection commences. *Communications Psychology* deposits these protocols in its own dedicated space, where they are public unless the authors request an embargo until the project is complete. This offers authors the opportunity to refer to a peer-reviewed version of the work before the data are even collected and to list this on their CVs. And it allows the journal to showcase the excellent peer-reviewed protocols entrusted to us.

Editors are acutely aware of how much the quality of peer-review matters. For Registered Reports, it can critically shape the outcome of the project. We are grateful for the trust that our authors place in us and looking forward to making a contribution to the success of future projects.
